# Treatment of blunt thoracic aortic injury in Germany—Assessment of the TraumaRegister DGU^®^

**DOI:** 10.1371/journal.pone.0171837

**Published:** 2017-03-27

**Authors:** Alexander Gombert, Mohammad E. Barbati, Martin Storck, Drosos Kotelis, Paula Keschenau, Hans-Christoph Pape, Hagen Andruszkow, Rolf Lefering, Frank Hildebrand, Andreas Greiner, Michael J. Jacobs, Jochen Grommes

**Affiliations:** 1 European Vascular Center Aachen-Maastricht, University Hospital RWTH Aachen, Aachen, Germany; 2 Klinikum Karlsruhe, Academic Teaching Hospital Univ. Freiburg, Karlsruhe, Germany; 3 Department of Orthopedic Trauma at Aachen University and Harald Tscherne Laboratory, University Hospital RWTH Aachen, Aachen, Germany; 4 Institute for Research in Operative Medicine (IFOM), University of Witten/Herdecke, Cologne, Germany; 5 Department of Vascular Surgery, University Hospital Charité Berlin, Berlin, Germany; Azienda Ospedaliero Universitaria Careggi, ITALY

## Abstract

**Purpose:**

Using the data delivered by the German Trauma Register DGU^®^ from 2002 till 2013, the value of different therapies of blunt thoracic aortic injury (BTAI) in Germany was analyzed.

**Methods:**

Prospectively collected data of patients suffering from BTAI were retrospectively analyzed with focus on the different treatment modalities for grade I–IV injuries.

**Results:**

821 patients suffering from BTAI were identified: 51.6% (424) grade I injury, 35.4% (291) grade II or III injury and 12.9% (106) grade IV injury (77.5% men [44.94 ± 20.6 years]). The main patterns of injury were high- speed accidents and falls (78.0% [n = 640], 21.8% [n = 171] respectively). Significant differences between grade I and grade II/III as well as IV injuries could be assessed for the incidence of cardiopulmonary resuscitation, a Glasgow Coma Scale score below 8 and a systolic blood pressure below 90 mmHg (p-value: <0.001). In the primary admission subgroup, 44.1% (197/447) of the patients received best medical treatment, 55.9% received surgical intervention (250/447): Thereof 37.2% (93/250) received open surgery and 62.8% (147/250) had been treated by endovascular means. Significantly lower 24-h- and in-hospital-mortality rates were encountered after endovascular treatment for all gradings of BTAI (p-value: <0.001). Yet this subgroup of patients showed the lowest incidence of further severe injuries and cardiac arrest.

**Conclusion:**

Endovascular therapy became the treatment of choice for BTAI in Germany. Patients who have been treated by surgical means showed the highest survival rate, especially endovascular therapy showed a favorable low mortality rate.

## Introduction

Blunt thoracic trauma is the second most common cause of thoracic aortic injury. The reasons are high-speed traffic accidents, explosions, fall from height and contusions [1]. 30% of all lethal accidents are caused by blunt thoracic aortic injury (BTAI). After post mortal examinations, up to 40% of all fatal accidents reveal a thoracic aortic injury as complication of a blunt thoracic trauma [2, 3]. In the group of seriously injured persons younger than 40 years, traumatic aortic rupture is frequently associated with further severe injury patterns [4]. Nearby 70–80% of the victims suffering from BTAI die on location. Within the first 24 hours, additional 30% of the surviving casualties will die because of aortic rupture or further live-threatening injuries. The incidence of blunt aortic injury is estimated between 1.5 and 2% of the patients suffering from blunt thoracic trauma [5].

The Society of Vascular Surgery established a classification system for blunt thoracic aortic injuries, depending on the grading of the aortic wall injury [6]. Grade I injury is characterized by an intimal tear, grade II injury is specified by an aortic wall hematoma, and grade III injury is determined by a pseudo aneurysm of the aortic wall. Grade IV injury indicates a free rupture of the thoracic aorta. This classification is based on CT- or MR-findings. Besides surgical therapy, conservative treatment has been described as an appropriate therapy for grade I and grade II injuries [7, 8]. If endovascular therapy is used, the minimal invasive character is a known benefit [9]. Additionally, further injuries of the affected patients can be treated simultaneously [10]. Single-center and multicenter studies demonstrated the feasibility of this technique with few post-procedural complications and reduced mortality rates [11, 12].

As a consequence, endovascular therapy of BTAI has become the treatment of choice within the last decade [13]. While there are registry studies in the US and other countries, the German national medical standard of care had not been analyzed and demonstrated yet [8]. The German society of vascular surgery (DGG) has published their guideline in 2008 and recommended endovascular treatment as therapy of choice. Till now, a nationwide treatment analysis of BTAI in Germany is pending. Hence we used the data, delivered by the Traumaregister DGU, to evaluate the pattern of treatment between the years 2002 till 2013.

## Materials and methods

### The TraumaRegister DGU^®^

The TraumaRegister DGU^®^, published by the German Trauma Society (Deutsche Gesellschaft für Unfallchirurgie, DGU) was founded in 1993. The aim of this multicenter database is a pseudonymous and standardized documentation of severely injured patients. It includes more than 63000 cases obtained from 615 trauma centers in Germany. Data is collected prospectively in four consecutive time phases, beginning at the site of the accident: A) Pre-hospital phase, B) Emergency room (ER) and initial surgery, C) Intensive care unit (ICU) and D) Discharge. Documentation includes detailed information on demographics, injury pattern, comorbidities, pre- and in-hospital management, course on intensive care unit, relevant laboratory findings including data on transfusion and outcome of each individual. Further details such as medication, pre-existing comorbidities and further details of the vascular injury are missing. The inclusion criterion is admission to hospital via emergency room with subsequent ICU care or reaching the hospital with vital signs. No follow-up data are provided on patients once they are discharged from the hospital after traumatic injury. The infrastructure for documentation, data management, and data analysis is provided by Academy for Trauma Surgery (AUC—Akademie der Unfallchirurgie GmbH), a company affiliated to the German Trauma Society. The scientific leadership is provided by the Committee on Emergency Medicine, Intensive Care and Trauma Management (Sektion NIS) of the German Trauma Society. The participating hospitals submit their data pseudonymously into a central database via a web-based application. Scientific data analysis is approved according to a peer review procedure established by Section NIS. The participating hospitals are primarily located in Germany (90%), but a rising number of hospitals of other countries contribute data as well (Austria, Belgium, China, Finland, Luxembourg, Slovenia, Switzerland, The Netherlands, and the United Arab Emirates). Currently, approximately 25,000 cases from more than 600 hospitals are entered into the database annually. The participation is voluntary. For hospitals associated with the TraumaNetzwerk DGU^®^, however, the entry of at least a basic data set is obligatory for reasons of quality assurance. This study followed the guidelines of the revised UN declaration of Helsinki in 1975 and its latest amendment in 1996 (42nd general meeting). There was no need for ethical approval. Furthermore, no informed consent was needed. The study was approved by the internal review board of the Section NIS. The present study is in line with the publication guidelines of the TraumaRegister DGU^®^ and registered as TR-DGU project ID. Injury distribution was determined according to the Abbreviated Injury Scale (AIS, version 2005) and the overall injury severity was summarized by the Injury Severity Score (ISS) [14] The AIS severity score ranges from 1 to 6 (1 = minor; 2 = moderate; 3 = serious; 4 = severe; 5 = critical; 6 = actual untreatable). For calculation of ISS each injury is allocated to one of six body regions: head and neck; face; chest; abdomen; extremities (including pelvis); and external. Only the highest AIS score in each body region was used. The three most severely injured body regions had their score squared and added to give the ISS. In order to assess the severity of traumatic brain injury the first pre-hospital Glasgow Coma Scale (GCS) was used in addition to the AIS score [15].

Clinical course included the length of stay on the intensive care unit, overall hospital stay and complications during hospital treatment such as sepsis or organ failure [16]. The diagnosis of sepsis was made according to the criteria of the ACCP/SCCM consensus conference committee [17, 18]. Organ function status was evaluated according to the Sequential Organ Failure Assessment (SOFA) score [19]. Organ failure was considered with 3 or more points an organ function, multiple organ dysfunction syndrome (MODS) was defined as simultaneous failure of at least two organs.

Inclusion criteria

The presented study included the following patients from the TR-DGU:

- Treated in a German trauma center level I
- Date of admission from January 2002 until December 2013
- Primary admission from the scene of injury (inter- hospital transfers excluded)
- Early transfer out (<48h) excluded since final outcome was not available
- Injury Severity Score (ISS) ≥ 16 points

**Fig 1:**
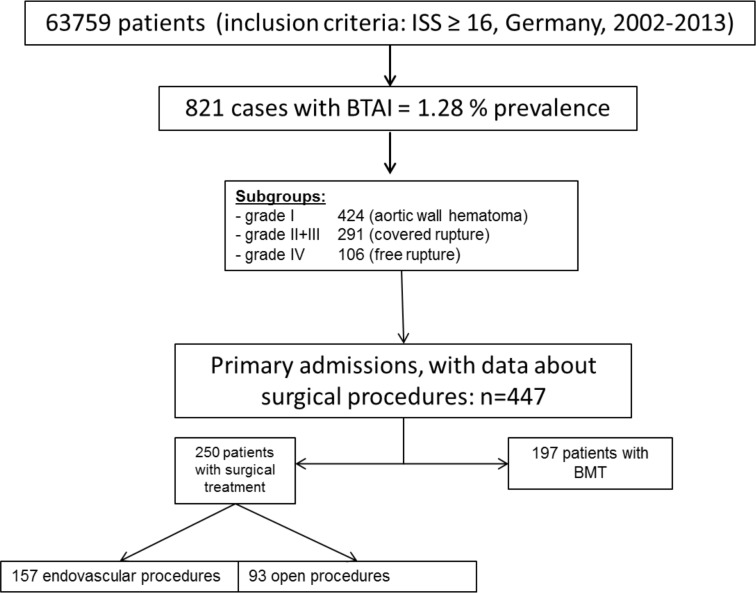
The structure of the patients’ selection based on the data delivered by the TraumaRegister. DGU^®^ from 2002 to 2013.

The whole structure of the patients selection can be seen in Fig 1.

The TraumaRegister DGU^®^ data from 2002 to 2013 was searched for “blunt thoracic aortic injury” following the International Statistical Classification terms (ICD). Based on the existing data set and the conditions of data collection of the Traumanetzwerk, a subdivision in three subgroups was possible: A separated analysis of grade II and III BTAI as recommended by the SVS was not possible, hence he following subgroups were formed:

1. Intimal tear
2. Aortic wall hematoma or pseudoaneurysm of the aortic wall
3. Open rupture

As a second step, the treatment modality (conservatively or operatively) was assessed. In this part only patients primarily treated in a level 1 trauma center were included because of superior data quality. Patients treated operatively were divided according to endovascular or open surgical intervention. An overview of all patient characteristics can be found in Table 1.

**Table 1:** Data of the pre- hospital and hospital phase and the mortality rate, separated by the three different groups of BTAI.

		total	grade I	grade II+III	grade IV	X^2^
**demographics**						
**number of all patients**	**% (n)**	**100 (821)**	**51.6 (424)**	**35.4 (291)**	**12.9 (106)**	
age	mean ± SD	44.9 ± 20.6	45.2 ± 20.3	44.9 ± 21.3	44.2 ± 19.9	
males	% (n)	77.5 (635)	75.5 (320)	79.6 (230)	80.2 (85)	0.371
**mechanism**	-	-	-	-	-	-
Motor vehicle collision	% (n)	41.3 (324)	44.3 (179)	38.4 (108)	37 (37)	
Motorcycle collision	% (n)	22.4 (176)	21.3 (86)	24.6 (69)	21 (21)	
Automobile vs. pedestrian or bicycle	% (n)	8.4 (66)	7.2 (29)	9.2 (26)	11 (11)	
Fall > 3m	% (n)	18.6 (146)	17.6 (71)	20.6 (58)	17 (17)	
Fall < 3m	% (n)	3.2 (25)	4.2 (17	2.5 (7)	1 (1)	
**pre-hospital phase**	-	-	-	-	-	-
Abdominal AIS > 3	% (n)	30.6 (251)	29.2 (124)	35.4 (103)	22.6 (24)	0.185
Extremity AIS > 3	% (n)	45.8 (376)	44.3 (188)	51.2 (149)	36.8 (39)	0.188
Head AIS > 3	% (n)	36.9 (303)	34.7 (147)	41.2 (120)	34 (36)	0.455
Blood transfusion	% (n)	47.3 (373)	39.4 (166)	54.2 (149)	63 (58)	**< 0.001**
more than 10 blood transfusions	% (n)	14.1 (111)	9.7 (41)	17.8 (49)	22.8 (21)	0.001
Transport via helicopter	% (n)	36.8 (213)	38.1 (103)	36,7 (80)	33 (30)	0.451
transfer time (min)	mean ± SD	64. 9 ± 26.9	66.0 ± 27.4	66.4 ± 27.6	57.9 ± 22.5	
**systolic bloodpressure < 90 mmHg**	**% (n)**	**46.3 (236)**	**35.6 (88)**	**50.5 (94)**	**70.1 (54)**	**< 0.001**
**Cardio- pulmonal resuscitation**	**% (n)**	**19.2 (114)**	**7.6 (21)**	**18.8 (42)**	**54.3 (51)**	**< 0.001**
**GCS < 8**	**% (n)**	**45.2 (255)**	**33.2 (87)**	**48.8 (105)**	**72.4 (63)**	**< 0.001**
**Intubation**	**% (n)**	**70.7 (420)**	**60.6 (168)**	**77.6 (173)**	**84 (79)**	**< 0.001**
**Need for catecholamine**	**% (n)**	**34.6 (153)**	**24.8 (50)**	**33.9 (59)**	**66.7 (44)**	**< 0.001**
Thoracic drain placement	% (n)	16.1 (71)	14.9 (30)	15.5 (27)	21.2 (14)	0.252
infusion volume pre- hospital (l)	mean ± SD	1.6 ± 1.1	1.4 ± 0.9	1.7 ± 1.3	1.7 ± 1.1	**<0.001**
**hospital phase**	-	-	-	-	-	-
systolic bloodpressure < 90 mmHg in ER	% (n)	44.3 (239)	31.5 (82)	50.3 (100)	70.4 (57)	**< 0.001**
blood transfusion	% (n)	49.6 (283)	42.1 (117	55.9 (118)	59.3 (48)	0.008
Cardio- pulmonal resuscitation	% (n)	28.5 (122)	9.7 (19)	31.3 (52)	76.1 (51)	**< 0.001**
ISS	mean ± SD	43.3 ± 18	33.8 ± 12.1	45.5 ± 14.1	75 ± 0	**< 0.001**
ICU stay (days)	mean ± SD	12.18 ± 15.2	14.74 ± 14.17	11.9 ± 17.34	2.71 ± 8.3	**< 0.001**
Intubation time (days)	mean ± SD	8.1 ± 12.3	9.4 ± 11.8	8.2 ± 13.9	2.29 ± 7.2	**< 0.001**
hospital length of stay (days)	mean ± SD	22.1 ± 24.8	28.1 ± 25.8	19.69 ± 23.7	4.5 ± 10.6	**< 0.001**
surgical treatment	mean ± SD	47.3 (389/821)	50.7 (215/424)	50.1 (146/291)	26.4 (28/106)	**< 0.001**
**mortality**	-	-	-	-	-	**-**
**24h mortality**						
**total**	**% (n)**	**34 (279)**	**13.4(57)**	**45(131)**	**85.8(91)**	**< 0.001**
medical treatment	% (n)	**49.1 (212)**	**20.6 (43)**	**66.9(97)**	**92.3(72)**	**< 0.001**
surgical treatment	% (n)	**17.2 (67)**	**6.5 (14)**	**23.3 (34)**	**67.9 (19)**	**< 0.001**
**in-hospital mortality**						
**total**	**% (n)**	**40.8 (335)**	**20 (85)**	**53.3 (155)**	**89.6 (95)**	**< 0.001**
medical treatment	% (n)	**56 (242)**	**28.7 (60)**	**75.2 (109)**	**93.6 (73)**	**< 0.001**
surgical treatment	% (n)	**23.9 (93)**	**11.6 (25)**	**31.5 (46)**	**78.6 (22)**	**< 0.001**

### Statistics

Incidences are presented with counts and percentages while continuous values are presented as mean and standard deviation (SD). Selected differences were evaluated using the chi- squared test for counts. A α of ≤.05 was used to demonstrate statistical significance The data was analyzed using the Statistical Package for the Social Sciences (SPSS; version 22; IBM Inc., Somers, NY, USA).

## Results

The German TraumaRegister DGU^®^ includes 63759 patients with an Injury Severity Score (ISS) > 16. Using the search term „blunt thoracic aortic injury“(BTAI) 821 cases were identified, which means a prevalence of 1.28%. 77.5% were men (n = 635). Mean age was 44.94 ± 20.6 years.

Based on the existing data set, these injuries were divided in three groups: subgroup 1 (51.6%, n = 424) included patients with an intimal tear; subgroup 2 (35.4%, n = 291) included those patients suffering from an aortic wall hematoma or a pseudo aneurysm. Subgroup 3 (12.9%, n = 106) included patients suffering from free rupture of the thoracic aorta.

All these patients reached the hospital alive and received imaging via CT- scan. Hypotension, defined as systolic blood pressure below 90 mmHg, was present in 46.3% (n = 236) of the patients. A Glasgow coma scale (GCS) below 8 was existent in 45.2% (n = 255) of all treated patients. The main patterns of injury were high- speed accidents involving cars, motorbikes and bicycles (78.0% [n = 640]). Other mechanism included were fall from height above 3 m (18.6%, n = 146) or below 3 m (3.2%, n = 25). Transport via helicopter was present in 36.8%, mean transfer time to the next hospital was 64.9 minutes. The scores for the abbreviated injury scale for abdomen, extremity and head above 3, indicating a serious injury of the affected region, amounted 30.6%, 45.8% and 36.9%. Regarding the AIS, no significant differences between the different gradings of BTAI could be shown. The 24-hours mortality rate was 34% (279/821), the in-hospital-mortality rate was 40.8% (335/821). More details can be found in Table 1.

The comparison of subgroup 1 and subgroup 2 as well a subgroup 1 and 3 revealed significant differences with regard to cardiopulmonary resuscitation (CPR), GCS, intubation, systolic blood pressure below 90 mmHg and the use catecholamine (p- value two-sided: < 0.001). The mean probability for blood transfusions was 47.3% (n = 373), with a significant difference between subgroup 1 (39.4%, [n = 166]) and subgroup 3 (63% [n = 58])(p- value two-sided: < 0.001). The mean Injury Severity Score (ISS) was 43.3 ± 18 with a significant difference between subgroup 1 (33.8 ± 12.1) and subgroup 3 (75 ± 0) (p- value two-sided < 0.001). Subgroup 3 (70.4% [n = 57]) displayed significantly more patients with hypotension than subgroup 1 (31.5% [n = 82]) and subgroup 2 (50.3% [n = 100]) CPR was performed in 28.5% (n = 122), with significant differences comparing subgroup 1 (9.7% [n = 19]), subgroup 2 (31.3% [n = 52] and subgroup 3 (76.1% [n = 51]).

### Surgical vs conservative treatment

In order to analyze the value of different treatment modalities, only patients primarily treated in level 1 trauma centers were included: By this, an improved data quality regarding the pre- hospital phase, the surgical treatment as well as the outcome on ICU (n = 447) could be guaranteed. In this group, 44.1% (n = 197) of the patients received best medical treatment (BMT) and 55.9% (n = 250) were treated by surgical means. 157 out of these 250 patients (62.8%) were treated by endovascular means and 93/250 (37.2%) patients received open surgery. Group 1 (patients with grade I BTAI) were treated by surgical means (n = 127) or conservative means (n = 76). In the group 3, 44 patients received conservative therapy and 23 surgical therapy. An AIS abdomen and extremity above 3 were more likely in the subgroup of patients receiving surgical repair. The conservative treatment cohort showed a significant increased rate of CPR as well as AIS head above 3 (p-value two-sided: <0.0001). Moreover a significant increased probability of a GCS below 8 could be seen in the group of conservatively treated patients (p-value two-sided: <0.0001). During the first 24 hours, the conservative treatment cohort displayed a significantly higher mortality rate (62.9% [124/197] in comparison to the surgical treatment cohort (23.2% [58/250], p- value two-sided < 0.001). The in-hospital-mortality rate was significantly higher after conservative treatment (71.5% [141/197] in comparison to the surgical treatment (29.6% [74/250], p- value two-sided < 0.001).

Particularly grade IV injuries with free rupture of the aorta displayed a high mortality independent of the kind of treatment: The in–hospital-mortality was 100% (44/44) after medical treatment and 86.5% (20/23) after surgical treatment. In contrast to the grade IV injuries, surgical treatment reduced the mortality significantly in subgroup 1 (grade I injury) and in subgroup 2 (grade II and III injuries). Subgroup 1 displayed a mortality rate of 44.7% (34/76) after conservative treatment and 12.5% (16/127) after surgical treatment. Subgroup 2 injuries showed a mortality rate of 85.7% (66/77) after BMT and 38% (38/100) after surgical treatment of BTAI (Table 2).

**Table 2:** Comparison of the conservative and surgical treatment subgroup.

		medical	surgical	X^2^
**total (n)**	**% (n)**	**44.1 (197)**	**55.9 (250)**	
grade I	% (n)	38.5 (76/197)	50.8 (127/250)	
grade II+III	% (n)	39 (77/197)	40 (100/250)	
grade IV	% (n)	23 (44/197)	9.2 (23/250)	
age (y, mean ±SD)	mean ± SD	46.4 ± 21.1	42.5 ± 18.8	0.041
gender (male, %)	% (n)	71.4 (140)	79.6 (199)	0.045
Abdominal AIS > 3	% (n)	13.9 (27)	17.7 (44)	0.28
Extremity AIS > 3	% (n)	39.6 (78)	53.6 (134)	0.003
**head AIS > 3**	**% (n)**	**51.3 (101)**	**33.6 (84)**	**<0.001**
**cardio- pulmonal resuscitation**	**% (n)**	**30.4 (59)**	**7.7 (19)**	**<0.001**
**GCS < 8**	**% (n)**	**59.7 (108)**	**35.9 (85)**	**<0.001**
intubation	% (n)	76.3 (148)	73.4 (182)	0.28
thoracic drain placement	% (n)	13.9 (27)	17.7 (44)	0.28
ICU stay (days)	mean ± SD	5.42 ± 3.21	13.64 ± 11.32	**<0.001**
Intubation time (days)	mean ± SD	3.74 ± 3.53	9.01 ± 4.21	**<0.001**
Hospital length of stay	mean ± SD	10.21 ± 7.64	22.08 ± 23.91	**<0.001**
**mechanism**				
traffic	% (n)	71 (132)	74 (182)	0.48
fall	% (n)	19.4 (36)	16.7 (41)	0.346
**24h mortality**		n = 197		
**total**	**% (n)**	**62.9 (124/197)**	**23.2 (58/250)**	
grade I	% (n)	36.1 (26/76)	8 (10/127)	
grade II+III	% (n)	75.3 (58/77)	31 (31/100)	
grade IV	% (n)	90.9 (40/44)	73.9 (17/23)	
**in-hospital mortality**				
**total**	**% (n)**	**71.5 (141/197)**	**29.6 (74/250)**	
grade I	% (n)	44.7 (34/76)	12.5 (16/127)	
grade II+III	% (n)	85.7 (66/77)	38 (38/100)	
grade IV	% (n)	100 (44/44)	86.9 (20/23)	

### Endovascular vs. open surgical treatment

In the subgroup of primarily in level I trauma centers surgically treated patients, 37.2% (n = 93) received open surgery and 62.8% (n = 157) endovascular repair. As we observed, more patients with severe aortic injuries were referred for open surgery than for endovascular therapy. Whereas two-thirds of the patients of subgroup 1 received endovascular therapy (65% [102/127]), we observed an equated distribution of both procedures in subgroup 2 (n = 49 vs n = 51 open vs endovascular). In line, most of the patients of subgroup 3 (19/23) were treated by open surgery (endovascular: 4/23). CPR was significantly more common in the open surgical subgroup (18.7% [n = 17] open vs. 1.3% [n = 2] endovascular, p-value two-sided: < 0.001). In contrast, severe injuries of the extremities and the head occurred more frequently in the endovascular subgroup, indicated by an AIS extremity > 3 of 63.1% (99/157) vs. 37.6% (35/93) as well as AIS head > 3 of 38.9% (61/157) vs. 24.7% (23/93) compared to the open surgical subgroup. The 24-hours–and the in-hospital-mortality showed a significantly decreased mortality rate in the endovascular treatment cohort (two-sided p-value < 0.001) (Table 3).

**Table 3:** Comparison of the open surgical and endovascular treatment subgroup.

		open	endovascular	X^2^
**demographics**		** **	** **	** **
**total (n)**	**% (n)**	37,2 (93)	62,8 (157)	
**grade I**	**% (n)**	26.9 (25)	65.0 (102)	**<0.001**
**grade II+III**	**% (n)**	52.7 (49)	32.5 (51)	**<0.001**
**grade IV**	**% (n)**	20.4 (19)	2.5 (4)	**<0.001**
age (y, mean ±SD)	mean ± SD	44.8 ± 19	41.2 ± 18.5	0.15
gender (male, %)	% (n)	77.4 (72)	80.9 (127)	0.50
**pre- hospital phase**				
Abdominal AIS > 3	% (n)	32.3 (30)	31.8 (50)	0.95
**Extremity AIS > 3**	**% (n)**	**37.6 (35)**	**63.1 (99)**	**< 0.001**
Head AIS > 3	% (n)	24.7 (23)	38.9 (61)	0.002
**Cardio- pulmonal resuscitation**	**% (n)**	**18.7 (17)**	**1.3 (2)**	**< 0.001**
GCS < 8	% (n)	47.1 (41)	29.3 (44)	0.006
Intubation	% (n)	80.2 (73)	69.4 (109)	0.064
Thoracic drain placement	% (n)	19.8 (18)	16.6 (26)	0.52
**mechanism**				
traffic	% (n)	63 (58)	80.3 (124)	0.003
fall	% (n)	17.4 (16)	16.2 (25)	0.461
**hospital phase**				
**Head AIS > 3**	**% (n)**	**24.4 (10)**	**38.0 (68)**	**< 0.001**
blood transfusion	% (n)	53.7 (22)	45.5 (81)	0.221
more than 10 blood transfusions	% (n)	12.2 (5)	11.8 (21)	0.561
acute renal failure	% (n)	15.6 (5)	9 (16)	0.197
sepsis	% (n)	18.2 (6)	13.6 (24)	0.327
multiorgan failure	% (n)	53.1 (17)	44.4 (79)	0.235
ISS	mean ± SD	31.17 ± 12	34.1 ± 10.5	0.298
ICU stay (days)	mean ± SD	11.93 ± 14.1	18.2 ± 14.3	0.187
intubation time (days)	mean ± SD	8.7 ± 12.6	11 ± 12.1	0.212
hospital length of stay	mean ± SD	24.6 ± 28.8	35.5 ± 26.7	0.178
**24h mortality**				
total	% (n)	26.8 (11)	1.7 (3)	< 0.001
**in- hospital mortality**				
total	% (n)	29.3 (12)	7.3 (13)	< 0.001

**Fig 2:**
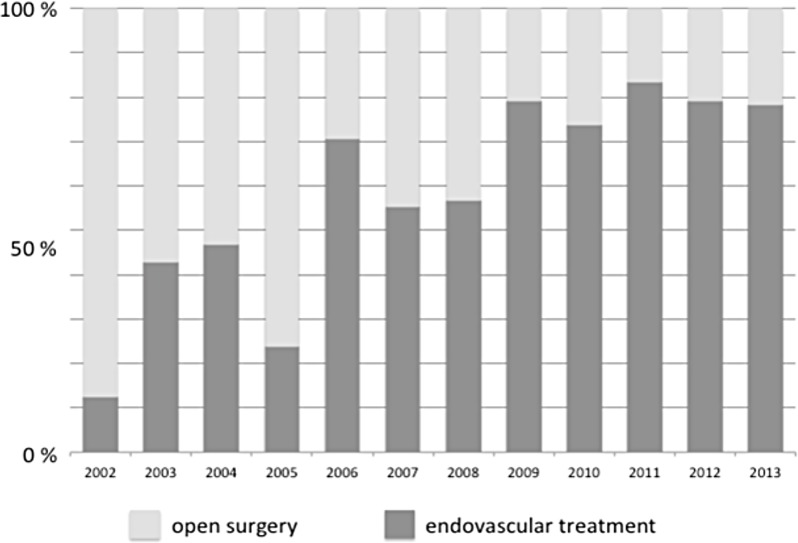
Development of endovascular and open surgical treatment of BTAI in Germany from 2002 till 2013.

Comparing the frequency of open surgical vs. endovascular treatment, a considerable trend towards endovascular therapy could be evaluated. Since 2006, an increasing number of patients had been treated by endovascular means. Finally 80% of all procedures were done by endovascular means in case of BTAI till 2013 (Fig 2).

## Discussion

Our study forms a representative survey of the treatment of BTAI in Germany. In accordance to the published literature, we observed an increased use of endovascular therapy compared with open surgery [13, 19]. Mortality rates, which correlate with the severity grade of BTAI, were significantly lower after surgical treatment except for grade IV injuries. Endovascular therapy showed favorable mortality and morbidity rates. However, a lot of patients with grade I injuries were treated by endovascular means. Hence, this pre-selection might over-estimate the reduction of mortality and morbidity by endovascular therapy.

Generally, patients suffering from grade IV injuries displayed a high mortality rate independently of the modality of treatment or additional existing injuries. Even grade II and III injuries displayed an increased mortality and morbidity rate compared to grade I injuries. Based on the available data, the injury pattern, evaluated by the abbreviation injury scale, is serious for all examined gradings of BTAI. No significant differences could be demonstrated, indicating a similarity of the severity of the non-aortic injuries, even if significant differences could be assessed regarding the rate of hypotension, CPR and the need of intubation. Regarding the pre- hospital phase, the grading of BTAI displayed a significant indicator for mortality. These findings match with the results presented by DuBose et al. in their retrospective multicenter trial [8]. Hypotension on admission, CPR, or the necessity of catecholamine therapy in the pre-hospital phase, as well as the mean ISS correlated with a higher mortality rate for grade II, III and IV injuries.

Interestingly, the mortality rate seems to be reduced by surgery in our patient cohort suffering from grade I, II and III injuries (subgroups 1 and 2). This observation is surprising, as the risk for rupture for BTAI grade I and II is generally estimated low and previous studies recommended medical treatment for patients suffering from grade I and II BTAI [7, 20]. Osgood et al. described a low progression rate of medical treated grade I and II BTAI within a follow up period of 86 days [20]. However, the patients in the present study who received medical treatment, displayed an AIS > 3 and a GCS <8 more frequently as well as a higher rate of CPR compared to those receiving surgical therapy. These findings indicate a poorer general condition of those patients, which partially explain the higher mortality rate in the conservative treatment subgroup. A conclusive explanation for the significant lower mortality rate in the surgical treatment group may be a quick decision for interventional or open surgical treatment independent from the present grading of BTAI, even if the studies mentioned above recommend a different strategy.

In addition it is conceivable, that patients, who suffered from severe injuries with an expected low probability to survive, received best medical treatment instead of surgical treatment.

We cannot dissolve this finding completely, because the TraumaRegister DGU^®^ did not provide the cause of death for each individual.

Consistent to the reduced mortality rate in the surgical treatment group, the length of stay on the ICU, as well as the in-hospital length of stay and the intubation time are significant longer in this subgroup. Furthermore, in contrast to previous studies, our survey showed considerable increased risk assessment scores for the pre-hospital phase for all subgroups. Du Bose et al. and Demetriades et al. demonstrated lower values for the ISS and for GCS as well as lower rates of hypotension on admission compared to this study [7, 8, 20]. In addition, a higher rate of conservative treatment for grade IV injuries was observed, which might be evaluated as palliative therapy for fatal injured persons, finally resulting in a higher mortality rate. This fact influenced the outcome of conservative treatment subgroup in the present study.

In 2007, Lettinga van Poll et al. and other authors demonstrated an improved survival rate after endovascular treatment of BTAI compared to open surgical therapy [13]. Nowadays, multiple single-center studies with numbers of patients between 7 and more than 50 demonstrated the benefits of the endovascular approach [21, 22]. If existing, a comparison with the open surgical treatment is mainly based on small numbers of patients. Lin et al. demonstrated their results in 2015, showing significantly improved outcome after endovascular vs. open surgical therapy of BTAI [23].

This study is able to compare similar endovascular and open surgical treatment subgroups. Significantly more patients suffering from grade I BTAI have been treated by endovascular means. Patients with grade II or III injuries were treated by open surgery and endovascular treatment nearly equally. The majority of patients suffering from grade IV injuries had been treated by open surgical therapy. Totally, only 23 patients suffering from grade IV BTAI have been treated surgically.

Patients treated by open surgery showed a significant increased rate of cardiac arrest in the pre-hospital phase. Hence a poorer general condition for this subgroup could be assumed. Moreover, the GCS as well as the frequency of AIS above 3 for abdomen and head are higher in the open surgery subgroup. Otherwise, all ICU- parameters showed reduced rates of complications in the endovascular subgroup, whereas no significant difference could be assessed while comparing open and endovascular treatment. Yet the mortality rates were significantly lower after endovascular treatment. These results may underline the value of endovascular therapy in case of BTAI. The analysis of the TraumaRegister DGU^®^ demonstrated a significant increase of endovascular therapy for BTAI in the last decade. Since 2011, more than 80% of all patients suffering from BTAI received endovascular therapy.

Based on to the character of the TraumaRegister DGU^®^, certain limitations have to be mentioned. Data was collected with focus on pre—and in- hospital management, course on intensive care unit and outcome of each individual. We were not able to analyze technical details of the endovascular and the open surgery. Moreover a separate analysis of the aortic wall hematoma and the pseudoaneurysm of the thoracic aorta would be preferable, which wasn’t possible because of the Trauma Register’s data collection. One further limitation is the missing possibility to separate aortic-related mortality from the general mortality rate. Moreover, risk factors and existing comorbidities were not available either. These facts reduce the validity of the presented information, as influencing factors could not be taken into account.

## Conclusion

Within the last decade, endovascular therapy became the treatment of choice for BTAI in Germany. Patients who have been treated by surgical means showed the highest survival rate, independent from the existing aortic injury grading. Especially endovascular therapy showed a favorable low mortality rate.

## Supporting information

S1 Fig Data extraction of the raw data delivered by the Traumaregistry. (DOCX)
