# Supplementary material for: Treatment of blunt thoracic aortic injury in Germany—Assessment of the TraumaRegister DGU®
Source: PLoS One. 2017 Mar 27;12(3):e0171837. doi: 10.1371/journal.pone.0171837 (PMC5367684; doi:10.1371/journal.pone.0171837)
Supplement: S1 Fig — (DOCX) [file pone.0171837.s001.docx]

**Supporting information**

Data extraction of the raw data delivered by the Traumaregistry

1. General information

Search term: Thoracic aortic injury 63759 including all patients

🡪 in hospital mortality: 18.6 all groups

IV: 20.0// V: 53.3 // VI: 89.6

🡪 24h mortality: 9.6 all groups

IV: 13.4// V: 45.0 // VI: 85.9

63759 patients with the search term BTAI

Further criteria: ISS > 16

Prevalence 1.6 in Traumaregistry

🡪 Mortality depends on the grade of BTAI

Comparison of the pattern of injury BTAI vs. trauma with ISS >16

| Pattern of injury | frequency (all traumata) | percent (%, all traumata) | frequency (BTAI) | percent (%, BTAI) |
| --- | --- | --- | --- | --- |
| Car accident | 16337 | 26 | 324 | 41,3 |
| motorcycle accident | 8306 | 13,2 | 176 | 22,4 |
| bicycle accident | 5207 | 8,3 | 22 | 2,8 |
| pedestrian | 5021 | 8 | 44 | 5,6 |
| fall > 3 m | 11624 | 18,5 | 146 | 18,6 |
| fall < 3 m | 11338 | 18 | 25 | 3,2 |
| other | 4987 | 7,9 | 0 | 0 |
| total | 62820 | 100 | 737 | 100 |
| Table 1: Frequency of different pattern of injuries leading to multiple trauma respectively BTAI (ISS >16) |  |  |  |  |

| Pattern of injury | | N | % | |
| --- | --- | --- | --- | --- |
| Vehicle accident | Car | 324 | 43.9 | 70.8 |
|  | Motorcycle | 176 | 23.9 |  |
|  | bicycle | 22 | 3 |  |
| Pedestrian | 44 | | 6 | |
| Fall > 3m | 146 | | 19.8 | |
| Fall > 3m | 25 | | 3.39 | |
| Total | 737 | | 99.99 | |

77.5 % male

72.5 % car and motorbike accident

II.

755 primarly treated patients subdivided in

- grade IV 398 (aortic wall haematoma)

- grade V 266 (covered rupture)

- grade VI 91 (free rupture)

Treatment in “Traumazentrum”

Level I // II // III

IV: 87.5 //10.8 // 1.7

V: 81.4 //17.2// 1.4

VI: 67.9// 29.2// 2.8

All: 82.8//15.5 // 1.7

Transfer to an level I “Traumzentrum”

IV: 33.7

V: 22.0

VI: 10.4

All: 26.6

Mean age: 44.94 years

Length of stay (all patients): 22.05 days

Length of stay (survivor): 28.06 days

Likelihood of thoracal drain placement:

IV: 14,9

V: 15.5

VI: 21.2

All: 16.1

Transport via chopper:

IV: 38.1

V: 36.7

VI: 33.0

All: 36.8

Intubation during transport:

IV: 60.6

V: 77.6

VI: 84.0

All: 70.7

CPR:
IV: 7.6

V: 18.8

VI: 54.3

All: 19.2

Catecholamines:

IV: 24.8

V: 33.9

VI: 66.7

All: 34.6

Systolic blood pressure below 90 mmHg:

IV: 35.6

V: 50.5

VI: 70.1

All: 46.3

Systolic blood pressure below 90 mmHg while arriving in the ER:

IV: 31.5

V: 50.3

VI: 70.4

All: 44.3

Blood transfusion pre-hospital:

IV: 42.1

V: 55.9

VI: 59.3

All: 49.6

CPR in the ER:

IV: 9.7

V: 31.3

VI: 76.1

All: 28.5

GCS below 8:

IV: 33.2

V: 48.8

VI: 72.4

All: 45.2

AIS Abdomen > 3:

IV: 29.2

V: 35.4

VI: 22.6

All: 30.6

AIS extremity > 3:

IV: 44.3

V: 51.2

VI: 36.8

All: 45.8

> 10 blood transfusions:

IV: 9.7

V: 17.8

VI: 22.8

All: 14.1

|  | IV (mean+SD+median) | V (mean+SD+median) | VI (mean+SD+median) | all (mean+SD+median) |
| --- | --- | --- | --- | --- |
| age | 45,1/20,3/44 | 44,8/21,3/42 | 44,23/19,9/43,5 | 44,9/20,6/44 |
| ISS | 33,8/12,1/32 | 45,4/14,1/43 | 75/0/75 | 43,3/18/41 |
| ICU days | 14,7/14,1/11 | 11,9/17,3/3 | 2,7/8,3/0 | 12,1/15,2/6 |
| intubation days | 9,4/11,8/4 | 8,2/13,9/1 | 2,2/7,2/0 | 8/12,3/2 |
| in hospital stay days | 28/25,8/22 | 19,6/23,7/9 | 4,5/10,6/1 | 22/24,8/16 |
| EK | 3,5/7,2 | 5,2/8,2/2 | 7,2/19,1/3 | 4,6/10,2 |
| volume pre-hospital (ml) | 1420/942/1250 | 1737/1268/1500 | 1653/1148/1375 | 1573/1112/1500 |
| transfer time | 66/27,4/62 | 66,3/27,6/63 | 57,9/22,5/55 | 64,9/26,9/61 |

Rate of endovascular treated patients:

2002 12.5

2003 42.9

2004 46.6

2005 23.8

2006 70.6

2007 55.2

2008 56.5

2009 78.9

2010 73.7

2011 83.3

2012 78.9

2013 78.3

III.

🡪 further analysis due to the follow-up and frame conditions only of the primarily treated patients: 447 patients

IV: 203

V: 177

VI: 67

Surgery: 250 (55.9%) vs. 197 (44.1%)

|  | IV | V | VI | all |
| --- | --- | --- | --- | --- |
| surgery vs BMT | 127 (62,6%) | 100 (56,5%) | 23 (34,3%) | 55,9 |
| endo | 80,3 | 51 | 17,4 | 62,8 |

No surgery (open or endo): death within 24h 68.1%

|  | best medical treatment (BTM) | surgical treatment | Odds ratio | 95 %CI | P Value |
| --- | --- | --- | --- | --- | --- |
| Death within 24h(n/%) | 124/ 62.9 | 58/23.2 | 0.18 | 0.12 – 0.27 | P <0.05 |

|  | IV | V | VI | all |
| --- | --- | --- | --- | --- |
| death within 24 h + surgery | 27,8 | 34,8 | 29,8 | 31,9 |
| death within 24h - surgery | 72,2 | 65,2 | 70,2 | 68,1 |
| survival > 24h with surgery | 70,1 | 78,4 | 60 | 72,5 |
| survical > 24h without surgery | 29,9 | 21,6 | 40 | 27,5 |
|  |  |  |  |  |
|  |  |  |  |  |
| 1. Without surgery, 68,1 BTAI-victims die < 24h |  |  |  |  |
| 2. With surgery, 31,9 BTAI-victims die < 24h |  |  |  |  |
| 3. With surgery, 72,5 BTAI-V survive 24h |  |  |  |  |
| 4. Without surgery, 27,5 survive 24h |  |  |  |  |

Surgical treated patients:

N = 250

Endo:

| IV | 19,70 | 80.3 |
| --- | --- | --- |
| V | 49.0 | 51.0 |
| VI | 82,59 | 17,11 |
| all | 37.2 | 62.8 |

Comparison BTAI treated open or endo

|  | open(mean+SD) | endo (mean+SD) | all (mean +SD) |
| --- | --- | --- | --- |
| age | 44,9 /21,02 | 40,8 /18,823 | 41,57 / 19,267 |
| ISS | 31,17 /12,058 | 34,17/10,508 | 33,61/10,84 |
| EK | 4,68 /10,86 | 3,51/ 5,99 | 3,73/ 7,14 |
| intubation days | 8,68 /12,62 | 11,02/ 12,18 | 10,58/12,2 |
| ICU days | 11,93 / 14,12 | 18,22/ 14,36 | 17,07 /14,4 |
| in hospital stay days | 24,66 /28,87 | 35,49/26,74 | 33,47/27,41 |

|  | open | endo | all |
| --- | --- | --- | --- |
| AIS head >3 | 24,398 | 38 | 35,5 |
| sepsis | 18,2 | 13,6 | 14,4 |
| Blood transf | 53,7 | 45,5 | 47 |
| > 10 Blood tranf | 12,2 | 11,8 | 11,9 |
| in hospital death | 29,3 | 7,3 | 11,4 |
| death < 24h | 26,8 | 1,7 | 6,4 |
| MOV | 53,1 | 44,4 | 45,7 |
| renal failure | 15,6 | 9 | 10 |

| IV | 101 | 26 |
| --- | --- | --- |
| V | 52 | 28 |
| VI | 4 | 19 |
| all | 157 | 93 |

|  | Death < 24h + Surgery | Death < 24h - Surgery | Odds ratio | 95% CI | P Value |
| --- | --- | --- | --- | --- | --- |
| IV | 10 | 26 | 0.25 | 0.11-0.55 | P <0.05 |
| V | 31 | 58 | 0.41 | 0.24-0.70 | P <0.05 |
| VI | 17 | 40 | 0.81 | 0.38-1.73 | 0.59 |

Complications

|  | Open (No.) | Endo (No.) | Odds ratio | 95% CI | P Value |
| --- | --- | --- | --- | --- | --- |
| AIS head >3 | 10 | 68 | 0.25 | 0.12-0.50 | P <0.05 |
| Sepsis | 6 | 24 | 0.42 | 0.17-1.07 | 0.07 |
| Blood transf. > 10 unit | 5 | 21 | 0.40 | 0.15-1.10 | 0.08 |
| In hospital death | 12 | 13 | 1.56 | 0.68-3.56 | 0.29 |
| Death < 24h | 11 | 3 | 6.19 | 1.68-22.76 | P <0.05 |
| MOV | 17 | 79 | 0.36 | 0.20-0.65 | P <0.05 |
| Renal failure | 5 | 16 | 0.53 | 0.19-1.49 | 0.23 |
